# Microbial Diversity Associated with the Cabernet Sauvignon Carposphere (Fruit Surface) from Eight Vineyards in Henan Province, China

**DOI:** 10.3390/foods13111626

**Published:** 2024-05-23

**Authors:** Junjie Zhang, Cancan Zhu, Zeyang Zhao, Chonghuai Liu

**Affiliations:** 1 Department of Biotechnology, College of Food and Bioengineering, Zhengzhou University of Light Industry, Zhengzhou 450002, China; zcc890986@163.com (C.Z.); 13271372851@163.com (Z.Z.); 2 Zhengzhou Fruit Research Institute, Chinese Academy of Agricultural Sciences, Zhengzhou 450009, China; liuchonghuai@caas.cn

**Keywords:** high-throughput sequencing, microbiota, metabolic function, Cabernet Sauvignon, grape surface, vineyard

## Abstract

The microbial diversity on the carposphere (berry) surface of the grape cultivar Cabernet Sauvignon grown in eight different locations/vineyards of Henan Province was determined by high-throughput sequencing of the bacterial 16S rRNA gene and fungal 18S rRNA gene. The structure of bacterial and fungal communities varied according to the sampling sites, but with some common phyla. Proteobacteria and Ascomycota were dominant/common phyla for bacteria and fungi, respectively. A total of 27 and 20 bacterial and fungal families, respectively, and 39 and 20 bacterial and fungal genera, respectively, with statistically significant differences, were found among different sampling sites. The difference for metabolic pathways of bacteria among the sampling sites existed. In addition, various abundances of enzymes from different sites might indicate that different function patterns exist in microbiota from different sites. The results revealed that locations of grape vineyards might play a significant role in shaping the microbiome, as well as the fact that vineyards can be distinguished based on the abundance of several key bacterial and fungal taxa. Overall, these findings extend our understanding of the similarities and differences in microbial community and their metabolic function on Cabernet Sauvignon grape surfaces from different geographic locations.

## 1. Introduction

Above and below ground plant surfaces, the phylloplane and rhizoplane, respectively, are specific habitats for microorganisms. Some of these microorganisms are pathogenic to plants and can inhibit their growth, but others can promote plant growth by, for example, suppressing pathogens, increasing nutrient availability, or influencing plant hormone balance [1]. For crop plants used in the food/feed and fermentation industries, surface microorganisms can play a role in determining the shelf life of products and influence the fermentation process [2,3,4,5].

Current methods used to assess microorganism diversity on plant surfaces are primarily based upon specific gene sequences. In particular, high-throughput sequencing of bacterial 16S rRNA and fungal 18S rRNA genes are carried out. These gene sequences can also be used to infer the metabolic and functional capabilities of the microorganisms. For example, for bacteria, PICRUSt (Phylogenetic Investigation of Communities by Reconstruction of Unobserved States test) updated to PICRUSt2 (http://github.com/picrust/picrust2, accessed on 1 August 2019), was developed for the prediction of functions from 16S rRNA data and is widely used [6]. Validation of PICRUSt2 matagenome predictions was carried out against seven wide-ranging published datasets generated using 16S rRNA sequences and shotgun metagenomics sequencing, providing a strong average correlation of 0.8 [6]. Nevertheless, care must be taken when inferring metabolic and functional capabilities of microorganism communities from ‘marker gene’ sequences.

Grape (*Vitis vinifera*) is grown primarily for its edible fruits, as well as the making of wine, which involves a fermentation process. Although most of the microorganisms on the grape surface cannot survive extreme conditions during the winemaking fermentation process, they can play an important role at the beginning of the process [7,8]). Previously, microorganisms associated with grape must (freshly crushed grape juice that contains skins, seeds and sometimes stem of the fruit) and the winemaking process have been extensively documented, but there are fewer studies of the microorganisms on the surface of the grape berry [9,10,11,12]. Zhang et al. [13] investigated the impact of grape variety and clone on grape berry surface bacterial communities at one vineyard in Henan Province, China. It was concluded that grape surface bacterial communities were affected by both grape variety and clone. Zhang et al. [14] assessed the diversity of non-*Saccharomyces* yeasts of grape berry surfaces from different Cabernet Sauvignon vineyards in Henan province. Here, it was concluded that geographic distribution and diversity of non-*Saccharomyces* yeast populations on Cabernet grape berries were likely to be determined by a combination of grape variety and environmental factors. The current study investigated the heterogeneity of microbial (bacterial and fungal) community associated with Cabernet Sauvignon in different vineyards of Henan Province. Cabernet Sauvignon is one of the world’s most widely cultured red wine grape varieties, and thus this work is of commercial as well as ecological importance.

## 2. Materials and Methods

### 2.1. Sample Collection, Transportation, and Storage

Grape samples were collected at 8 vineyards in the ancient riverbed of the Yellow River wine grape region in Henan Province, China. The sample sites were Anyang (AY), Zhengzhou (with two vineyards, ZNPA and ZPZ), Minquan (MQ), Fugou (FG), Changyuan (CY), Wugang (WG), and Luohe (LH), which are mainly distributed in the north and middle of the province (Figure S1 and Table S1). For each site, the vineyard was randomly selected from the main grape planting region, and three samples as replicates were collected from each vineyard. For each replicate, three bunches of healthy grape berries from a random selected grape plant were sheared and stored temporarily in sterilized plastic bags on ice for immediate transport to the laboratory. For all sites, samples were taken one week before the maturation of grapes during August and September 2017 (Table S1). In addition, the grape peels with minimum grape flesh were taken immediately under aseptic condition after arriving at the laboratory. The grape peels of each replicate were placed in individual sterilized tubes and stored at −80 °C until further processing.

### 2.2. DNA Extraction and PCR Amplification

Microbial DNA was extracted from grape peel samples using the Fast DNA SPIN Kit for Soil (MP) according to the manufacturer’s protocols. The final DNA concentration and purity were determined by NanoDrop 2000 UV-VIS spectrophotometry (Thermo Scientific, Wilmington, NC, USA), and DNA quality was checked by 0.8% (*w*/*v*) agarose gel electrophoresis. The V3-V4 hypervariable regions of the bacterial 16S rRNA gene were amplified with primers 338F (5′-ACT CCT ACG GGA GGC AGC AGA-3′) and 806R (5′-GGA CTA CHV GGG TWT CTA AT-3′) [15], and the fungal 18S rRNA gene was amplified with primers of SSU0817F (5′-TTA GCA TGG AAT AAT RRA ATA GGA-3′) and 1196R (5′-TCT GGA CCT GGT GAG TTT CC-3′) [16] using a thermocycler PCR system (GeneAmp 9700, ABI, Shanghai, China). The PCR reactions were conducted as follows: 3 min of denaturation at 95 °C; 27 cycles of 30 s at 95 °C for denaturation, 30 s for annealing at 55 °C, and 45 s for elongation at 72 °C; then, a final extension at 72 °C for 10 min. PCR reactions were performed in triplicate with a 20 μL mixture containing 4 μL of 5 × FastPfu Buffer, 2 μL of 2.5 mM dNTPs, 0.8 μL of each primer (5 μM), 0.4 μL of FastPfu Polymerase, and 10 ng of template DNA. The resulting PCR products were extracted from a 2% (*w*/*v*) agarose gel after electrophoresis and further purified by using the AxyPrep DNA Gel Extraction Kit (Axygen Biosciences, Union City, CA, USA) and quantified using QuantiFluor™-ST (Promega, Beijing, China) according to the manufacturer’s protocol.

### 2.3. Illumina Miseq Sequencing Analysis

The purified amplicons were pooled in equimolar and paired-end sequenced (2 × 300) forms on an Illumina MiSeq platform (Illumina, San Diego, CA, USA) according to the standard protocols by Majorbio Bio-Pharm Technology Co., Ltd. (Shanghai, China). The raw reads were deposited into the NCBI Sequence Read Archive (SRA) database under the accession numbers PRJNA707343 for bacteria and PRJNA707314 for fungi. Raw fastq files were demultiplexed, quality-filtered by Trimmomatic, and merged by FLASH with the following criteria: (i) the reads were truncated at any site receiving an average quality score <20 over a 50 bp sliding window; (ii) primers were exactly matched allowing 2 nucleotide mismatching, and reads containing ambiguous bases were removed; (iii) sequences whose overlap was longer than 10 bp were merged according to their overlap sequence. Operational taxonomic units (OTUs) were clustered with 97% similarity cutoff by the SILVA database [17] and web-based tools [18]. Richness, Shannon index, ACE index, Chao1, and Coverage were included in the alpha diversity analysis by using the MOTHUR program [19]. The heat map for relative abundances of different taxonomic groups was constructed by R software version 3.3.1 (www.r-project.org/, accessed on 1 August 2019). The unweighted pair-group method with arithmetic mean (UPGMA) was used to calculate the beta diversity index, based on the phylogenetic tree used to compute phylogenetic relatedness of the bacterial and fungal communities between samples. The vegan package version 1.0.8 of R software (Version 3.1.2) was used to calculate the community similarity based on OTUs using non-metric multidimensional scaling (NMDS) analysis based on Bray–Curtis distance matrices and ANOSIM analysis. For taxonomic annotations, sequences of the obtained non-redundant gene catalog were annotated against the NCBI NR database, a non-redundant protein database, using BLASTP (BLAST Version 2.2.28+, http://blast.ncbi.nlm.nih.gov/Blast.cgi, accessed on 1 August 2019) with an e-value cutoff of 1 × 10^−5^. Searches against the KEGG (Kyoto Encyclopedia of Genes and Genomes) database were conducted for functional annotation. The relative abundance of different functional hierarchy was equal to the sum of relative abundance of genes annotated to that functional level.

### 2.4. Statistical Analysis

Data were analyzed using the Microsoft Excel 2016 and Origin 2017 (Origin Lab, Guangzhou, China) programs on Microsoft Windows and presented as means with standard deviation of treatments. One-way analysis of variance (ANOVA) was performed to investigate the statistical significance of the relative abundance of organisms within the microbial community using SPSS analysis (Statistical Product and Service Solutions 24.0 Windows, SPSS Inc., Chicago, IL, USA) and corrected by Duncan test (*p* < 0.05 as the significant threshold). The vegan package in R software (Version 3.1.2) was used to calculate the community similarity based on OTUs using non-metric multidimensional scaling (NMDS) for analysis of similarity (ANOSIM) based on the Bray–Curtis distance matrix. The relative abundance of different functional hierarchy was equal to the sum of relative abundance of genes annotated to that functional level. The level 3 KEGG ortholog functions of the relative abundance of the main 50 metabolic functions were drawn on a heatmap using the vegan package of R software (Version 3.1.2). A linear discriminant analysis effect size (LEfSe) was applied to the OTU table (non-parametric factorial Kruskal–Wallis sum-rank test *p* < 0.05, LDA > 3.0) to identify the discriminant bacterial clade, using the Huttenhower Galaxy web application with the LEfSe algorithm (http://huttenhower.sph.harvard.edu/galaxy/, accessed on 1 August 2019).

## 3. Results

### 3.1. Sequence Analysis

A total of 1,389,109 bacterial sequences and 873,020 fungal sequences were obtained for the 24 samples from the eight vineyards sampled. Average sequence length was 429 and 402 base pairs, and the average sequence number was 57,880 and 20,952 per sample for bacteria and fungi, respectively. A total of 19 phyla, 38 classes, 86 orders, 154 families, 281 genera, 387 species, and 494 OTUs for bacteria and 7 phyla, 18 classes, 31 orders, 39 families, 41 genera, 50 species, and 61 OTUs for fungi were identified from the samples.

The diversity indices of grape peel microbial communities (bacteria and fungi) are presented in Table 1. For bacterial communities, the Shannon index for site WG was similar to that for site ZPZ but greater than that for all other sites. Also, the Shannon index for site ZPZ was greater than that for LH but was not significantly different from those of the other sites. For fungal communities, the Shannon index for any one site was not significantly different from at least two other sites.

### 3.2. Evolution of Microbial Communities

NMDS analysis for both bacterial and fungal communities based on the three replicates for the different sites partitioned the different samples into three distinct groups (Figure S2). With two exceptions, the bacterial communities in the triplicate samples from the same sites grouped together (Figure S2a). The sampling sites of LH, AY, and CY clustered in group one; WG and FG in group two; and ZPZ, ZNPA, and MQ group three. The two exceptions were MQ-3 and ZNPA-2, both of which were in group 1. For fungal communities, AY, ZNPA, ZPZ, and MQ formed a cluster; WG, LH, and FG formed another cluster; and CY and FG-1 formed a third (Figure S2b). Although the groups for bacteria and fungi showed some similarities, e.g., WG and FG, and ZPZ and ZNPA grouped together in both, there were substantial differences between them. In particular, for bacteria, LH, CY, and AY formed into a cluster while they were distributed into three different groups on analysis for fungi.

At the phylum level, diversified dominancy of phyla was observed in different sampling sites (Figure S3a,b). In the bacterial community (Figure S3a), Cyanobacteria was the dominant phylum (relative abundance 59.25% to 95.28%) for all the sampling sites, which possibly included chloroplasts. Proteobacteria (2.28% to 29.70%) and Actinobacteria (1.49–8.15%) were the second and third abundant phyla, respectively, in all the sampling sites, while Bacteroidetes, Epsilonbacteraepta, etc., were site-specific phyla (Figure S3a). Conversely, in the fungal community (Figure S3b), the Ascomycota phylum was dominant for all sampling sites ranging from 77.64% for LH to 98.15% for CY. Basidiomycota was prevalent in all sites, while LH presented the highest content (22.36%), and CY was the least (1.68%). In ZNPA, ZPZ, and WG, the phylum Ciliophora was also detected with abundance more than 1.0%. The results demonstrated Proteobacteria, Actinobacteria, Ascomycota, and Basidiomycota as the core microbial phyla across the sampling sites on the Cabernet Sauvignon grape surface.

At the class level (Figure S3c,d), all the sampling sites had the same dominant bacterial class of Oxyphotobacteria, ranging from 59.22% for ZPZ to 91.78% for AY, consistent with those at the bacterial phylum level (Figure S3a). The bacterial class Alphaproteobacteria presented in ZPZ, MQ, and WG with the relative abundances of 11.79%, 10.80%, and 10.52%, respectively, while it presented in other sites in abundance less than 7.0%. The relative abundance of Gammaproteobacteria varied from 17.57% and 13.00% in ZPZ and FG, respectively, to less than 8.00% in the remaining sites. Actinobacteria presented in FG and WG with abundances of 8.15% and 7.11%, respectively, and less than 5.00% in the remaining sites (Figure S3c). In Figure S3d, the fungal class Dothideomycetes was dominant in AY, ZNPA, ZPZ, MQ, and WG, ranging from 56.91% in WG to 84.20% in ZNPA, while in the remaining three sites (FG, CY, and LH), it occupied 16.67%, 36.55%, and 34.19%, respectively. The fungal class Sordariomycetes occupied 65.60–12.21% in FG, CY, LH, WG, AY, and MQ, and it was less than 10% in the remaining sites. The class Exobasidiomycetes presented in LH and CY with the relative abundance of 22.08% and 16.59%, respectively, while it was less than 10% in all the remaining sites.

**Figure 1:**
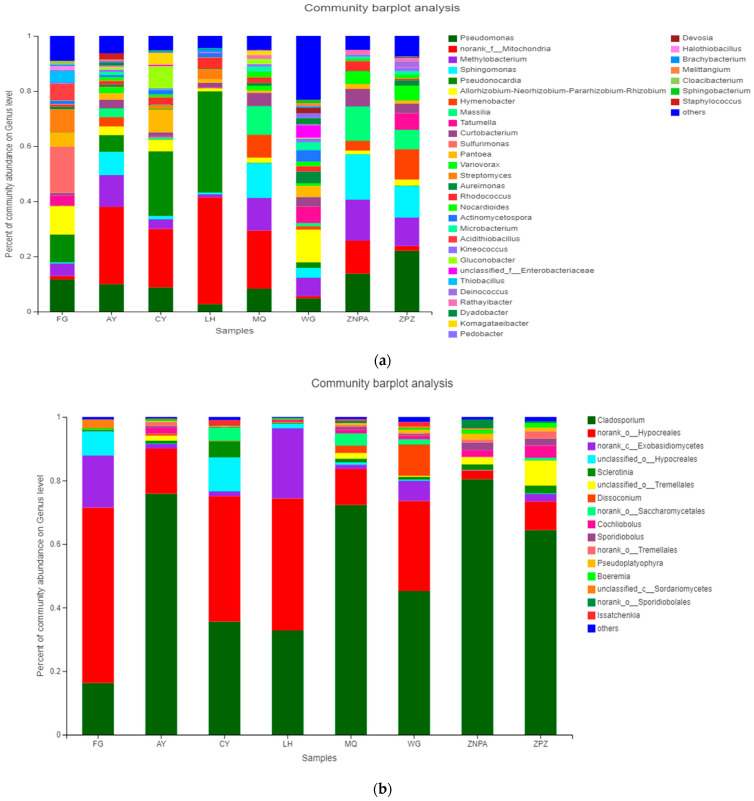
Taxonomic structure of microbiota at the genus level, (**a**) for bacteria and (**b**) for fungi.

At the genus level (Figure 1a), all the sampling sites had the same dominant bacterial genus of norank_o__Chloroplast ranging from 59.22% for ZPZ to 95.27% for LH, demonstrating it as the common genus on the Cabernet Sauvignon grape surface. For the bacterial genus of *Pseudomonas*, ZPZ had the highest content of 9.0%, followed by FG with 4.11%. For *Methylobacterium*, ZPZ also had the highest content of 4.22%, while MQ and WG had 2.40% and 2.03%, respectively. In addition, ZPZ also had the highest contents of the following genera: *Sphingomonas*, *Hymenobacter*, *Massilia*, *Tatumella*, *Curtobacterium,* and *Variovorax*. And FG had the highest contents of the following genera: *Pseudonocardia*, *Allorhizobium-Neorhizobium-Pararhizobium-Rhizobium*, *Pantoea,* and *Streptomyces.* Conversely, in Figure 1b, *Cladosporium* and g__norank_o __Hypocreales were the top two fungal genera in all the samples, with the contents ranging from 16.29% to 80.41% and from 2.71% to 55.18%, respectively. ZNPA had the most abundance of *Cladosporium,* while FG had the least of 16.29%. And FG had the most abundance of g__norank_o__Hypocreales, but ZNPA only contained 2.71%. FG and LH had the high contents of g__norank_c__Exobasidiomycetes at 16.42% and 22.07%, respectively. CY had the most abundance of g__unclassified_o__Hypocreales at 10.71%. FG also had the highest content of *Sclerotinia* at 5.14%. ZPZ had 7.89% of g__unclassified_o__Tremellales, and WG had 9.83% of *Dissoconium.* ZPZ also had the highest contents of *Cochliobolus* (3.85%), *g__norank_o__Tremellales* (2.3%), and *Boeremia* (1.38%). ZNPA had the most contents of *Sporidiobolus* (2.37%), *Pseudoplatyophyra* (1.93%), and g__norank_o__Sporidiobolales (2.73%). CY, FG, and WG had the most contents of the following genera of g__norank_o__Saccharomycetales (4.17%), g__unclassified_c__Sordariomycetes (2.78%), and *Issatchenkia* (1.58%), respectively.

### 3.3. Differences in Bacterial and Fungal Diversity among Vineyards

**Figure 2:**
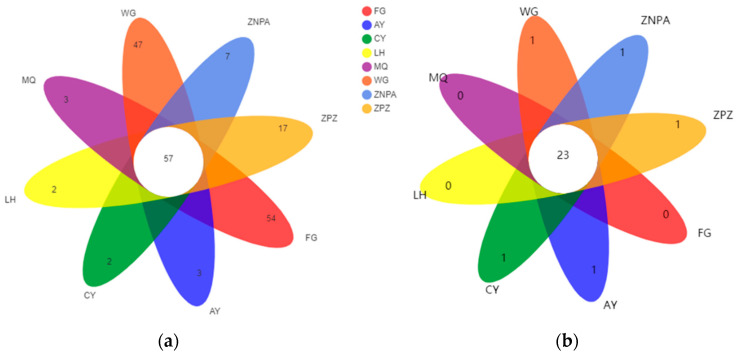
Venn diagrams of operational taxonomic units (OTUs) at cut-off 0.03 for the bacterial (**a**) and fungal (**b**) communities on grape surface in different sampling sites.

Comparison of the bacterial OTUs shared among different sample sites showed that the unique OTUs varied from 54 in FG to 2 in CY and LH, while 57 OTUs were shared in all of the eight sampling sites that took up 9.6% of the total OTUs (Figure 2a). Compared to that of the bacterial communities, the fungal OTUs shared among different sample sites were much less (Figure 2b). The following sites of WG, ZNPA, ZPZ, AY, and CY were only with one unique OTU, and there was no unique OTUs of FG, LH, and MQ. And the shared OTUs of the eight sampling sites were 23 in number, which took up 37.7% of the total OTUs (Figure 2b).

**Figure 3:**
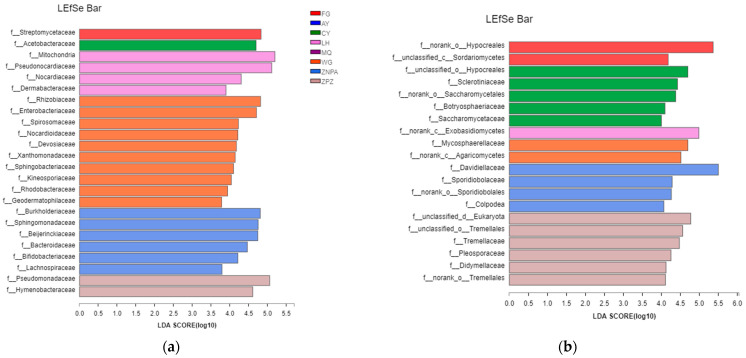
Taxonomic differences at family level among grape surface bacteria (**a**) and fungi (**b**) in different sampling sites by a linear discriminant analysis (LDA) effect size (LEfSe) method, where the LDA threshold was 2.0.

**Figure 4:**
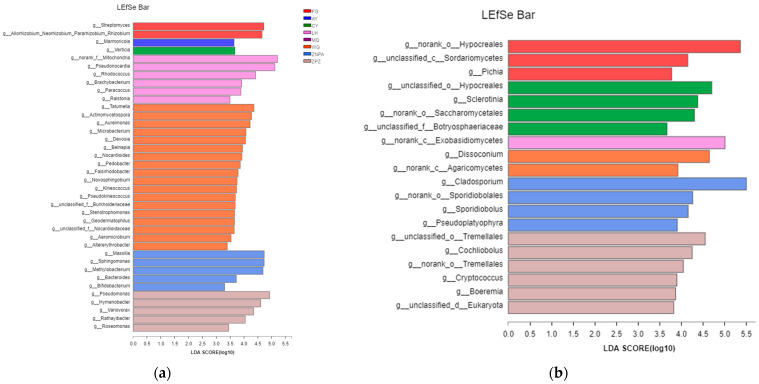
Taxonomic differences at the genus level among grape surface bacteria (**a**) and fungi (**b**) in different sampling sites by a linear discriminant analysis (LDA) effect size (LEfSe) method, where the LDA threshold was 2.0.

To determine the classified taxa with significant abundance differences among the sampling sites, a biomarker analysis based on the linear discriminant analysis (LDA) effect size (LEfSe) method was performed (Figure 3). With an LDA threshold of 2.0, there were 24 and 20 bacterial and fungal families and 38 and 20 bacterial and fungal genera with statistically significant differences among the sampling sites, respectively. The abundances of 16 bacterial families were different between WG and ZNPA sites, followed by WG and LH with 14 families (Figure 3a). Enriched bacterial families were ten in WG, six in ZNPA, four in LH, two in ZPZ, and one in FG and AY, while no biomarker was detected in AY and MQ (Figure 3a). It was shown that most of the sampling sites could be clearly distinguished by the demonstrative microorganism at the family level. In addition, the greatest difference of bacterial genera was also obtained between WG and LH (24 genera), including 18 genera in WG and 6 genera in LH (Figure 4a).

Conversely, the fungal community composition of ZPZ and CY had the greatest difference of 11 fungal families (Figure 3b). Six families in ZPZ, five families in CY, four families in ZNPA, two families in FG, two families in WG, and one family in LH were significantly enriched. Again, no biomarker was observed in AY and MQ. It was shown that most samples from different sites could be clearly distinguished by the demonstrative microorganism by the relative abundances of fungal families. In addition, the greatest difference of fungal genera was also obtained between ZPZ and CY or ZNPA (10 genera) (Figure 4b).

In order to elucidate the differences in bacterial and fungal compositions among different vineyards, the Bray–Curtis dissimilarity was adopted to study the most common wine grape in China, Cabernet Sauvignon, which is also common in the world, and it was analyzed independently to dissect inter-vineyard biogeographical relationships. The patterns in grape surface microbiota suggest a genetic component to host–microbial interactions on the grape surface. We calculated the taxonomic metric (Bray–Curtis dissimilarities calculated from OTU), and the tests showed that community structure varied among different vineyards, exerting a significant impact of bacterial Bray–Curtis on the grapes among vineyards (*R^2^*_adonis_ = 0.590, *p* < 0.01; *R*_anosim_ = 0.633, *p* < 0.01). For the fungal Bray–Curtis, tests showed that community structure varied among different vineyards, exerting a significant impact of fungal Bray–Curtis on the grapes among vineyards (*R^2^*_adonis_ = 0.857, *p* < 0.01; *R*_anosim_ = 0.766, *p* < 0.01) (Table S2). These results revealed that grape vineyards play a significant role in shaping bacterial and fungal community in the phyllosphere, while vineyards can be distinguished based on the abundance of several key bacterial and fungal taxa.

**Figure 5:**
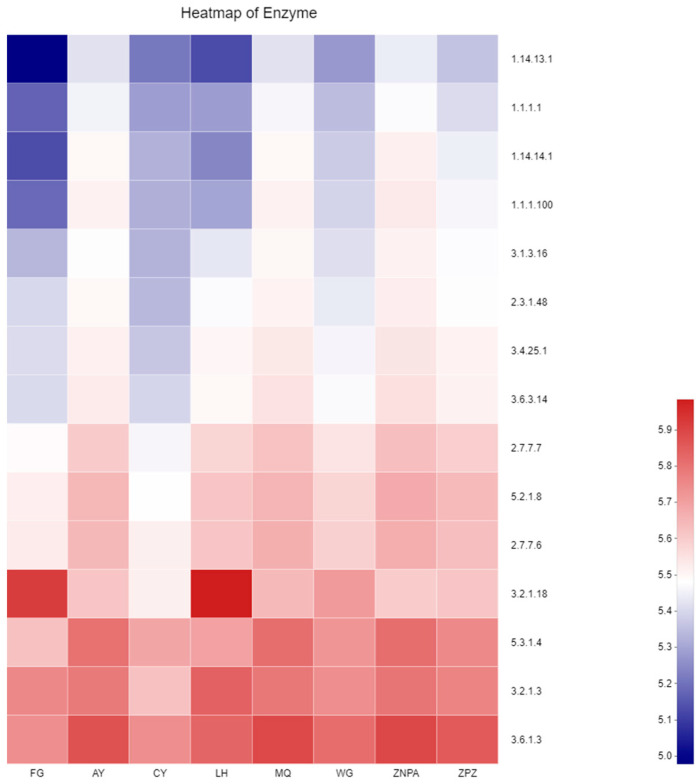
Variation of fungal enzyme profiles of different sampling sites by PICRUSt2.

Conversely, for fungal analysis, the predicted gene sequences annotated with the KEGG pathway in the 24 samples corresponded to 851 different enzymes in total (Figure 5). The most abundant enzymes included adenosinetriphosphatase, L-arabinose isomerase, glucan 1,4-alpha- glucosidase, etc., and the abundances of these enzymes varied in different sites that might demonstrate different function compositionin different sites.

## 4. Discussion

In previous studies on grapes in vineyards in Henan Province, China, it was concluded that grape surface bacterial communities were affected by grape variety and clone, while geographic distribution and diversity of non-*Saccharomyces* yeast populations on Cabernet Sauvignon grape berries were likely to be determined by a combination of grape variety and environmental factors [13,14]. In the current study, the heterogeneity of bacterial and fungal communities associated with Cabernet Sauvignon in different vineyards of Henan Province was investigated. Here, the Shannon Index for bacterial and fungal communities on grape berries could differ between sites but, generally, there was considerable overlap of values across sites, and further work (greater replication, different times of sampling) is required to establish if site differences are consistent.

### 4.1. Difference of Epiphytic Bacterial and Fungal Taxa among the Vineyards

Comparison of the microbial communities on wine grape surface from eight sampling sites showed that the dominant phyla, classes, and genera were common in all sites, but their abundance varied in different sites. Of all vineyards, the class Dothideomycetes was predominant in five sites, while Sordariomycetes was dominant in the remaining three sites. In addition, classes like Alphaproteobacteria, Gammaproteobacteria, and Actinobacteria had a higher abundance in some sites, involving FG, MQ, WG, and ZPZ, while class Campylobacteria was only found in FG (Figure S3c). Though norank_o_Chloroplast was the dominant bacterial genus in all the sites, ZPZ had the least content of 59.22%, and LH had the most of 95.27%, which indicated about 36% difference among the sampling sites and suggested that it appeared strongly influenced by region [20]. ZPZ had the highest contents of *Pseudomonas*, *Methylobacterium*, *Sphingomonas*, *Hymenobacter*, *Massilia*, *Tatumella*, *Curtobacterium*, and *Variovorax*, which might have resulted from ZPZ being sampled from a grape garden with hundreds of grape varieties and clones, where the microbiota on the grape surface might have been much more complex than those from the vineyard planting only one single variety of grape. Furthermore, *Cladosporium* was the dominant fungal genus but with contents ranging from 16.29% to 80.41% from the eight sampling sites, which suggested a huge differentiation among different vineyards. g__norank_o__Hypocreales was the second most abundant genus prevalent in all sites, while the contents were with a wide range of 2.71% to 55.18%. Interestingly, the revealed phyla of bacteria as well as fungi were positively relevant to winemaking [21]. According to the results, Cyanobacteria phylum relative abundance was found to be 59.25% to 95.28%, being dominant in all the sampling sites. Cyanobacteria are well known for the large-scale synthesis of products of interest due to their easier growth requirements and ability to capture solar energy as well as fixing of carbon dioxide [22]. Moreover, Cyanobacteria biomass has the storage of glycogen and is also used as carbohydrates as well as nutrient feedstock for the production of bioethanol [23,24]. Furthermore, the second and third abundant phyla Proteobacteria (2.28% to 29.6%) and Actinobacteria (1.49–8.15%) were also reported for encompassing spoilage and fermenting species [21]. Similarly, in fungi, Ascomycota was found dominant, which is also well established in vineyards and wineries anthropogenically [25]. The overall results could suggest that although the samples were from different sites, they contained some common microbial groups, being strongly influenced by their original regions, which is similar to a previous report [20].

Furthermore, 27 and 20 bacterial and fungal families were significantly different from the different sampling sites, respectively. The greatest difference of 18 bacterial families of the bacterial communities between WG and ZPZ was found, and WG and 14 different families were found between FG and WG (Figure 3a). And 11 fungal families were found to be the greatest between ZPZ and CY. In addition, various amounts of bacterial and fungal families were significantly enriched from the different sampling, sites and the eight sampling sites could be distinguished by grape surface microbiota at the family level. In addition, 39 and 20 bacterial and fungal genera with statistically significant differences were found among different sampling sites, respectively. A total of 31 bacterial genera were found significantly enriched between WG and ZPZ, and 10 fungal genera were significantly different between ZPZ and CY or ZNPA. Furthermore, AY and MQ did not even have common families and genera with significant abundance both for bacteria and fungi. This suggests that though the samples were all from the vine variety of Cabernet Sauvignon, they might enrich different kinds of microbes from their growing local environments, management, and farming activity, which is consistent with former reports [20,26].

### 4.2. Influence of Vineyards on the Metabolic Functions of Epiphytes on Grape Berries

KEGG analysis displayed the 6764 Kos, in which 45 small metabolic pathways and 323 metabolic subsystems were revealed for bacterial communities on grape surfaces. This is positively correlated with the vineyards health and attributed grape surfaces that might be useful to fight against various type of disease and ecological maintenance. The identified pathways are mostly involved in six basic metabolic systems, namely, metabolism, genetic information processing, environmental information processing, cellular processes, human diseases, and organismal systems, while the abundance of metabolism was more than other pathways.

During fungal analysis, the three most abundant enzymes were found to be adenosine triphosphatase, glucan 1,4-alpha- glucosidase, and L-arabinose isomerase. Interestingly, the enzyme activity might play a principal role in vineyard sustainability and wine quality, which affects the fermentation process at before, during, and after the must fermentation stages, respectively. The adenosine triphosphatase abundance was positively related to vineyard sustainability because the uptake of monovalent metal cations by the grape plant roots and the influx of these ions into tissue as well as leaf were directly influenced the cropping level, water status, rootstock, and nutrition of minerals. Furthermore, the above related characteristics and potentiality directly affected the pH and titratable acidity of grape juices [27]. Glucan 1,4-alpha- glucosidase is important in wine production as it works as a sensor for wine quality. And it is the main technological property of yeast strain must that is possessed [28]. Overall, the abundances of enzymes from different sites were different, which might result from the biogeographic distributions involving the climate, temperature, and microbiota from the local farms. Finally, it indicated the different functions on grape surfaces among the vineyards.

## 5. Conclusions

In this study, different microbiota and their metabolic function on the Cabernet Sauvignon grape surface from eight vineyards in Henan Province were analyzed. The results showed that different sampling sites caused differences in bacterial and fungal community structures. Cyanobacteria and Ascomycota were the dominant phyla for bacteria and fungi, respectively. This indicated the common microbial phylum composition from different sampling sites, but also different compositions from different sites were detected. A total of 27 and 20 bacterial and fungal families and 39 and 20 bacterial and fungal genera with statistically significant differences were found among different sampling sites, respectively. The difference for metabolic pathways of bacteria among the sampling sites existed. In addition, various abundances of enzymes from different sites might indicate different function composition from different sites. Overall, the findings extend our understanding of differences of microbial community and their metabolic function on Cabernet Sauvignon grape surface from different geographic distributions.

## Figures and Tables

**Table 1:** Alpha diversity of bacteria and fungi communities on surface of Cabernet Sauvignon grape in different vineyards.

Sample ID	Bacteria *				Fungi *			
	Shannon	Simpson	Ace	Chao1	Shannon	Simpson	Ace	Chao1
AY	3.11 ± 0.60 ab	0.13 ± 0.09 a	160.51 ± 25.73 ab	161.86 ± 41.36 ab	1.08 ± 0.18 a	0.55 ± 0.06 c	44.61 ± 6.92 c	40.33 ± 1.53 b
CY	2.65 ± 0.43 ab	0.17 ± 0.10 a	136.23 ± 19.37 ab	141.27 ± 23.23 ab	1.57 ± 0.20 bcd	0.28 ± 0.05 a	43.25 ± 1.50 c	43.36 ± 0.88 b
FG	2.98 ± 0.32 ab	0.10 ± 0.03 a	189.00 ± 47.32 bc	188.24 ± 47.71 bc	1.30 ± 0.13 abc	0.37 ± 0.05 ab	31.37 ± 1.35 a	30.62 ± 0.66 a
LH	1.94 ± 0.86 a	0.35 ± 0.26 a	104.46 ± 23.93 a	98.17 ± 25.24 a	1.42 ± 0.06 abc	0.31 ± 0.04 a	34.15 ± 3.50 ab	36.64 ± 7.95 ab
MQ	2.61 ± 1.64 ab	0.30 ± 0.42 a	145.36 ± 43.29 ab	148.18 ± 30.72 ab	1.28 ± 0.44 ab	0.51 ± 0.18 bc	41.41 ± 3.02 c	41.75 ± 4.40 b
WG	4.40 ± 0.03 c	0.02 ± 0.00 a	300.10 ± 36.74 d	301.20 ± 34.22 d	1.78 ± 0.19 d	0.26 ± 0.06 a	42.25 ± 2.38 c	41.87 ± 2.58 b
ZNPA	2.85 ± 0.64 ab	0.14 ± 0.12 a	116.57 ± 43.05 a	120.48 ± 35.66 a	1.09 ± 0.06 a	0.61 ± 0.02 c	31.07 ± 3.00 a	30.83 ± 3.25 a
ZPZ	3.52 ± 0.93 bc	0.08 ± 0.05 a	239.46 ± 16.95 c	239.95 ± 20.96 c	1.71 ± 0.25 cd	0.36 ± 0.10 a	39.57 ± 4.02 bc	39.83 ± 4.86 b

*. Coverages were >0.98818 for bacteria and >0.99973 for fungi in all the samples. Different letters following the numbers in each column indicate significant difference in the Duncun test (*n* = 3, *p* = 0.05) by ANOVA analysis.

## Data Availability

The original contributions presented in the study are included in the article/Supplementary Materials, further inquiries can be directed to the corresponding author.

## Supplementary Materials

The following supporting information can be downloaded at: https://www.mdpi.com/article/10.3390/foods13111626/s1, Figure S1: Distribution of the sampling sites in Henan Province.; Figure S2: Non-metric multidimensional scaling analysis for the bacterial communities based on the Bray–Curtis similarity index; Figure S3: Taxonomic structure of microbiota at the phylum (3a and 3b) and class level (3c and 3d).; Table S1: Location and climate information of the sampling sites; Table S2: Adonis and Anosim factors revealing effects of sampling sites on microbial diversity patterns (n = 3).
