# Supplementary material for: Microbial Diversity Associated with the Cabernet Sauvignon Carposphere (Fruit Surface) from Eight Vineyards in Henan Province, China
Source: Foods. 2024 May 23;13(11):1626. doi: 10.3390/foods13111626 (PMC11172321; doi:10.3390/foods13111626)
Supplement: Supplementary file 1 [file foods-13-01626-s001.zip › foods-2961770-Supplementary figures .pdf]

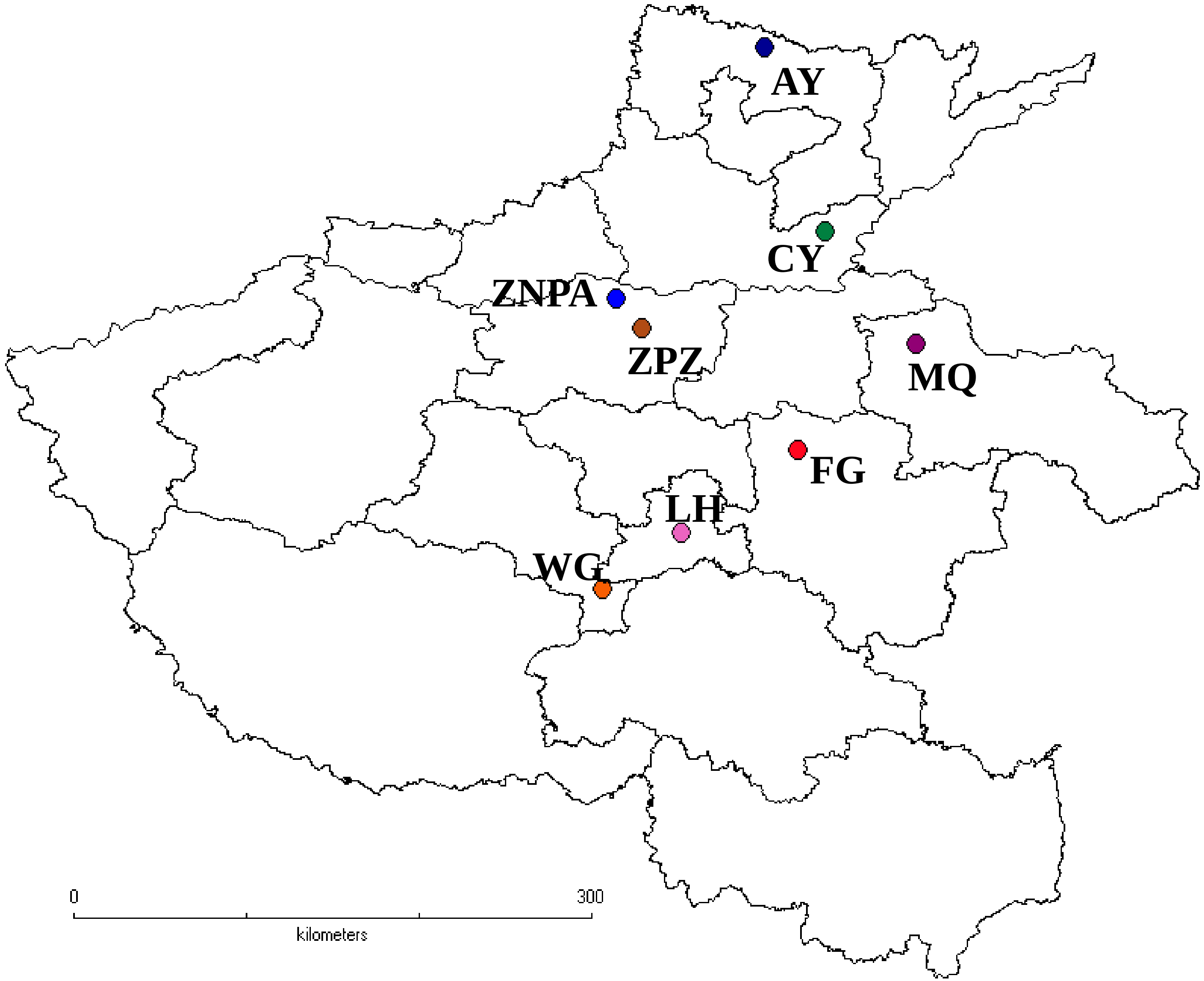

Suppl. Fig. S1 Distribution of the sampling sites in Henan Province. ZPZ and ZNPA from Zhengzhou, AY from Anyang, FG from Fugou, Zhoukou, WG from Wugang, Pingdingshan, LH from Luohe, CY from Changyuan, Xinxiang and MQ from Minquan, Shangqiu.

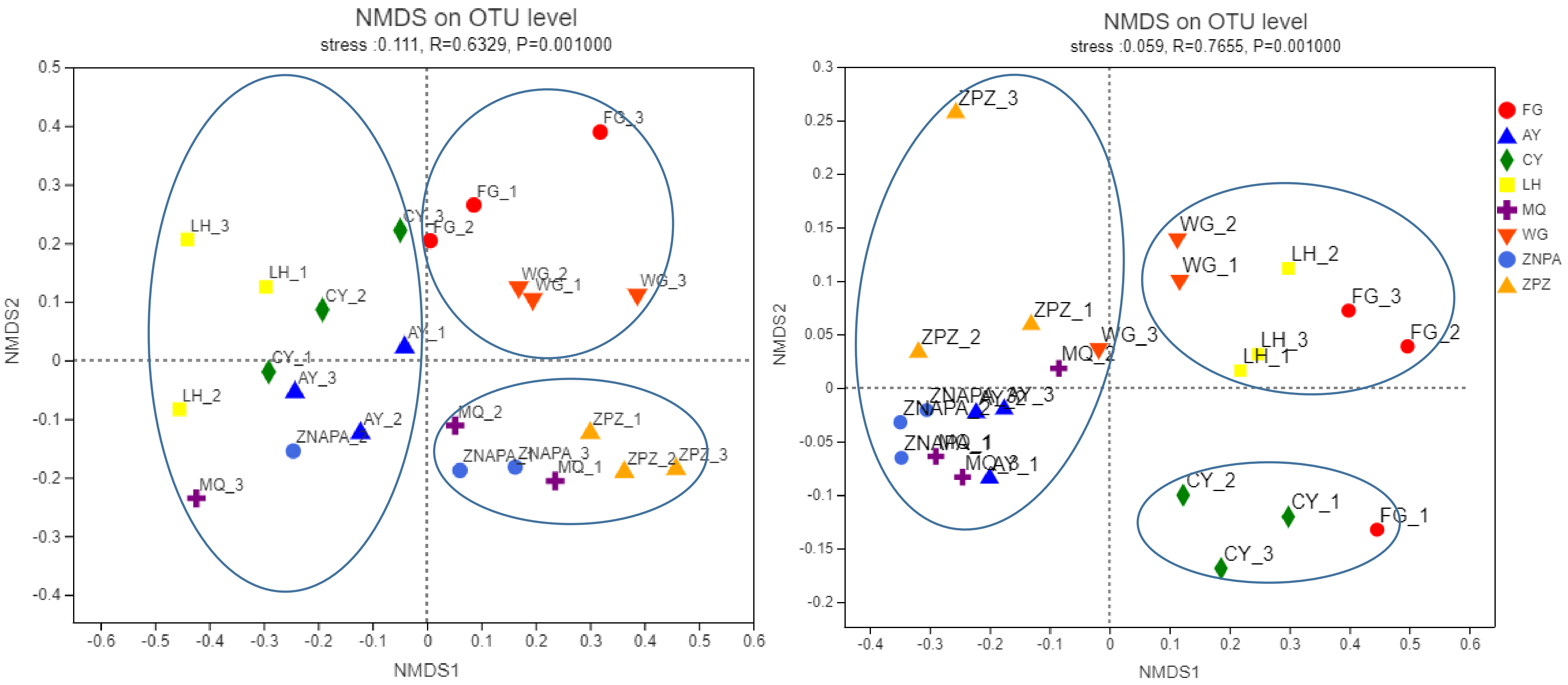

S2a S2b

Suppl. Fig. S2 Non-metric multidimensional scaling analysis for the bacterial communities based on the Bray–Curtis similarity index. a for bacteria and b for fungi.

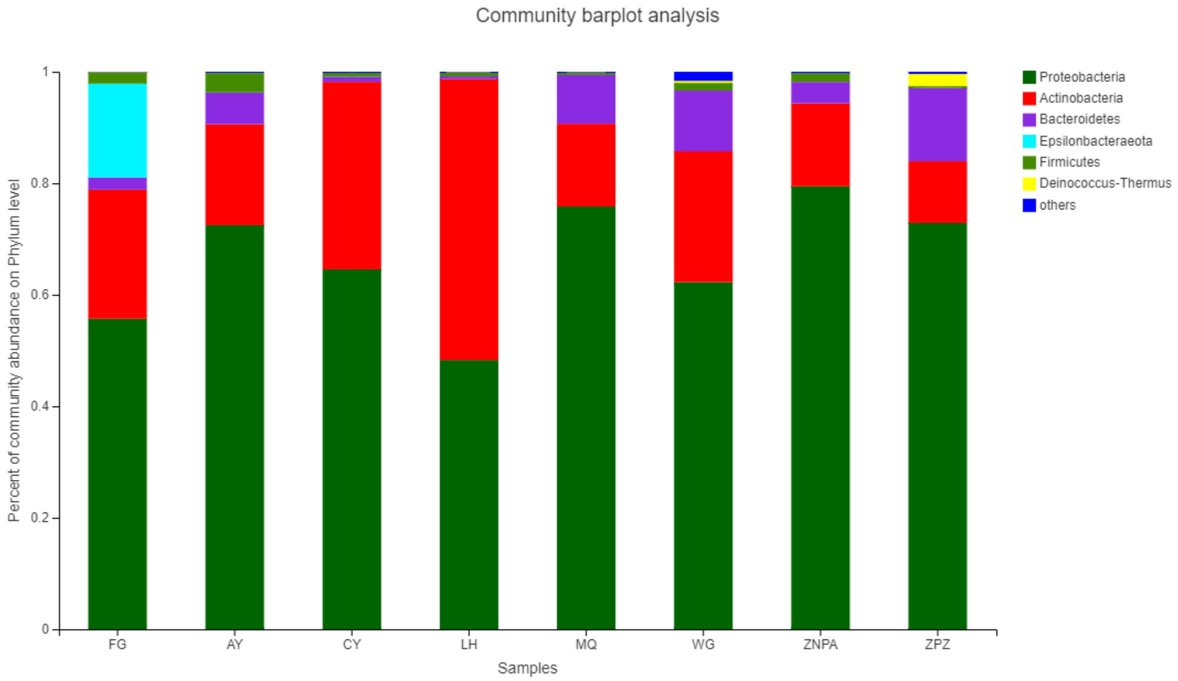

S3a

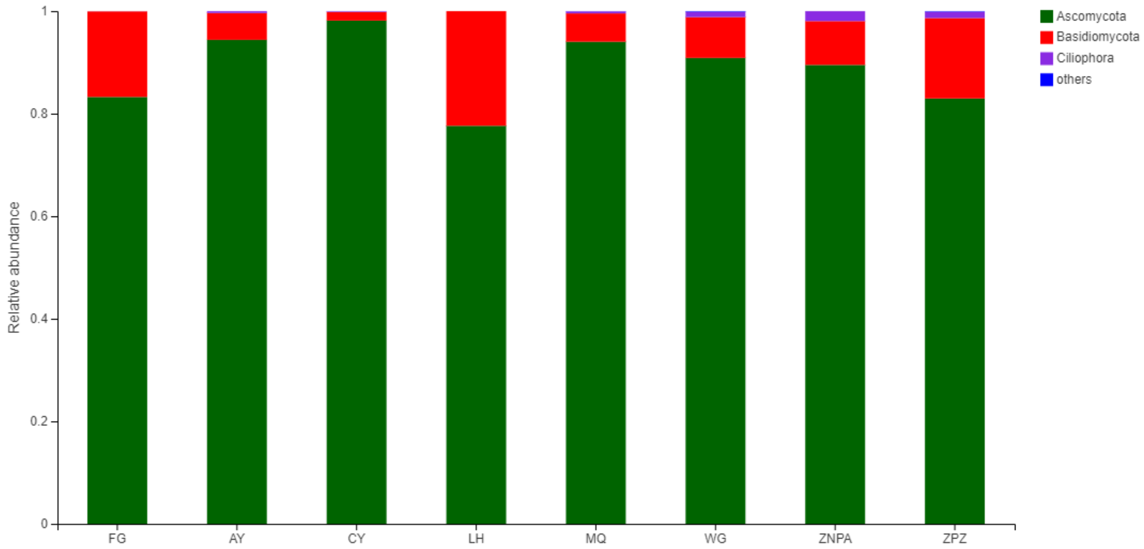

S3b

Suppl. Fig. S3 Taxonomic structure of microbiota at the phylum (3a and 3b) and class level (3c and 3d). And 3a and 3c for bacteria and 3b and 3d for fungi.

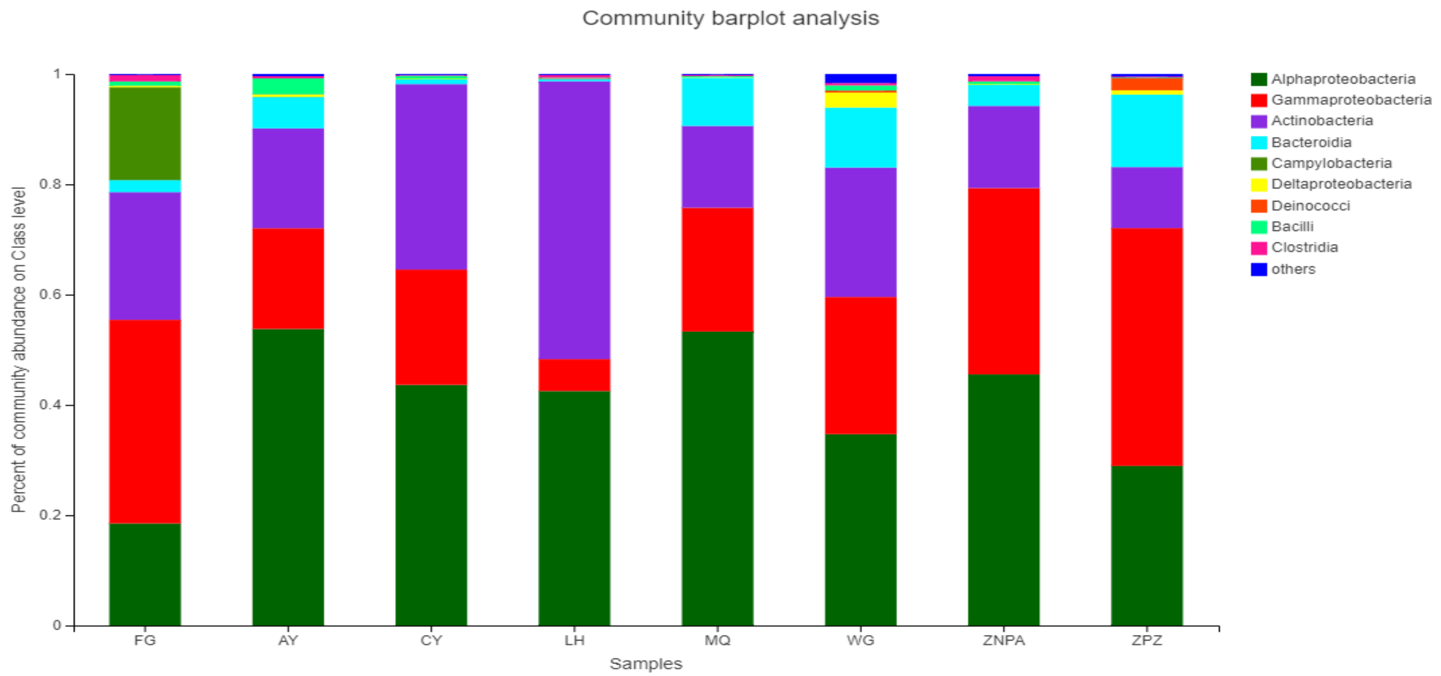
Community barplot analysis
Percent of community abundance on Class level
Samples
FG
AY
CY
LH
MQ
WG
ZNPA
ZPZ
Alphaproteobacteria
Gammaproteobacteria
Actinobacteria
Bacteroidia
Campylobacteria
Deltaproteobacteria
Deinococci
Bacilli
Clostridia
others

S3c

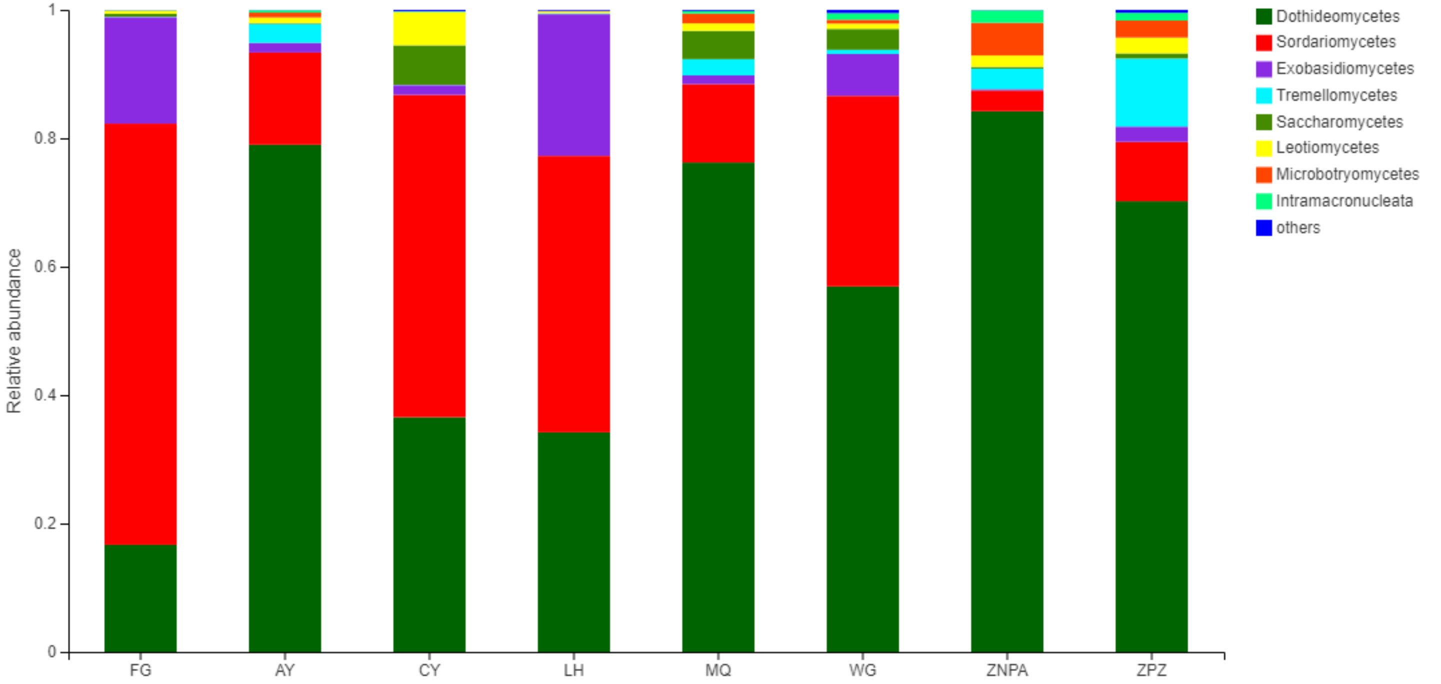
Relative abundance
FG
AY
CY
LH
MQ
WG
ZNPA
ZPZ
Dothideomycetes
Sordariomycetes
Exobasidiomycetes
Tremellomycetes
Saccharomycetes
Leotiomycetes
Microbotryomycetes
Intramacronucleata
others

S3d
