# Supplementary material for: Microbial Diversity Associated with the Cabernet Sauvignon Carposphere (Fruit Surface) from Eight Vineyards in Henan Province, China
Source: Foods. 2024 May 23;13(11):1626. doi: 10.3390/foods13111626 (PMC11172321; doi:10.3390/foods13111626)
Supplement: Supplementary file 1 [file foods-13-01626-s001.zip › foods-2961770-Supplementary Tables.pdf]

Table S1. Location and climate information of the sampling sites*

Sample ID	Vineyard	Location	Sampling time	Altitude (m)	Tmin (℃)	Tmax (℃)	Precipitation (mm/yr)
**AY**	Anyang	N36.1492, E114.3575	Sep. 29,2017	75	15.8	27.6	55
**CY**	Changyuan	N35.2076, E114.6732	Sep. 20,2017	63	16.1	27.3	57
**FG**	Fugou	N34.0832, E114.5334	Sep. 30,2017	57	16.1	26.7	70
**LH**	Luohe	N33.6626, E113.9155	Sep. 19,2017	63	16.3	26.6	76
**MQ**	Minquan	N34.6265, E115.1502	Sep. 20,2017	62	16.1	26.7	66
**WG**	Wugang	N33.3788, E113.5011	Sep. 19,2017	86	16.5	26.7	83
**ZNPA**	Zhengzhou Napa	N34.8618, E113.5692	Sep. 01,2017	100	15.8	26.7	83
**ZPZ**	Zhengzhou Ziyuanpu	N34.7132, E113.7039	Agu.25,2017	104	21.6	30.8	122

*. Climate data of the sampling sites were acquired from DIVA-GIS (http://www.diva-gis.org)

Table S2. Adonis and Anosim factors revealing effects of sampling sites on microbial diversity patterns (n=3)

Bacterial Bray-Curtis				Fungal Bray-Curtis			
ADONIS		ANOSIM		ADONIS		ANOSIM	
R^2	p	R	p	R^2	p	R	p
0.590	0.001	0.633	0.001	0.857	0.001	0.766	0.001
